# Disaster Response: Mental Health Effects among WTC Rescue and Recovery Workers

**Published:** 2008-09

**Authors:** Carol Potera

The attacks on the World Trade Center (WTC) on 11 September 2001 exposed thousands of emergency responders and other recovery workers to a unique mix not only of airborne toxic pollutants but also psychological stressors. The physical consequences such as persistent respiratory ailments have been documented previously [e.g., *EHP* 114:1853–1858 (2006)]. The latest report from a 5-year study of health effects among WTC rescue and recovery workers describes a higher level of lingering mental health problems among these workers than in the general population **[*EHP* 116:1248–1253; Stellman et al.]**.

More than 10,000 WTC workers completed several standard mental health questionnaires 10–61 months after the attacks. About 90% of the respondents worked at the WTC site during the first 2 weeks after 9/11, and the majority remained onsite for 3 months or longer. On the basis of an analysis of their responses, and in the absence of a clinical evaluation, the researchers classified 11.1% of workers with probable post-traumatic stress disorder (PTSD), 8.8% with probable depression, 5.0% with probable panic disorder, and 62% with substantial stress reactions (such as nightmares, flashbacks, and insomnia). Overall, mental health problems declined gradually from 13.5% to 9.7% among WTC workers during the course of the study.

The incidence of PTSD in WTC workers, which parallels that reported in soldiers returning from combat duty in Afghanistan, was about 4 times higher than that for the general population in the United States. Probable PTSD was associated with having lost family members or friends in the attacks; those with probable PTSD had a 17-fold greater likelihood of reporting disruption of family, work, and social life. About half those with probable PTSD also experienced probable panic disorder, depression, or both. Workers with probable PTSD also perceived their children as having more psychological symptoms (such as clinginess or trouble sleeping) and behavioral problems than workers without PTSD.

Alcohol-related problems also were abundant in the study group. More than 17% reported symptoms of probable alcohol abuse. Nearly half reported drinking more heavily than usual during the period they worked at rescue and recovery efforts, and months later a third were still drinking more than usual.

The authors conclude that the variety of persistent mental health problems in responders “underscores the need for long-term mental health screening and treatment programs targeting this population.” Following future environmental disasters, they write, mental health problems are virtually certain to accompany physical effects of toxic exposures. Rescue and recovery workers therefore should receive behavioral health evaluations as well as medical evaluations to reduce adverse health and social consequences.

## Figures and Tables

**Figure:**
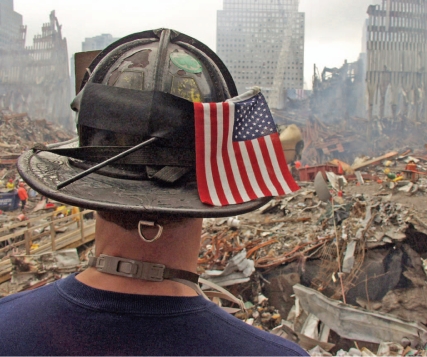
A worker surveys the WTC site, 25 September 2001

